# mRIN for direct assessment of genome-wide and gene-specific mRNA integrity from large-scale RNA-sequencing data

**DOI:** 10.1038/ncomms8816

**Published:** 2015-08-03

**Authors:** Huijuan Feng, Xuegong Zhang, Chaolin Zhang

**Affiliations:** 1 MOE Key Laboratory of Bioinformatics and Bioinformatics Division, TNLIST/Department of Automation, Tsinghua University, Beijing 100084, China; 2 Department of Systems Biology, Department of Biochemistry and Molecular Biophysics, Center for Motor Neuron Biology and Disease, Columbia University, New York, New York 10032, USA

## Abstract

The volume of RNA-Seq data sets in public repositories has been expanding exponentially, providing unprecedented opportunities to study gene expression regulation. Because degraded RNA samples, such as those collected from post-mortem tissues, can result in distinct expression profiles with potential biases, a particularly important step in mining these data is quality control. Here we develop a method named mRIN to directly assess mRNA integrity from RNA-Seq data at the sample and individual gene level. We systematically analyse large-scale RNA-Seq data sets of the human brain transcriptome generated by different consortia. Our analysis demonstrates that 3′ bias resulting from partial RNA fragmentation in post-mortem tissues has a marked impact on global expression profiles, and that mRIN effectively identifies samples with different levels of mRNA degradation. Unexpectedly, this process has a reproducible and gene-specific component, and transcripts with different stabilities are associated with distinct functions and structural features reminiscent of mRNA decay in living cells.

## Abstract (web summary)

With the rapid increase in the volume of publically available RNA-seq data, quality control is an increasingly important consideration. Here Feng *et al*. develop mRIN, a method to directly assess mRNA integrity, and show that RNA degradation in post-mortem samples has a strong impact on global expression profiles.

## Graphical abstract

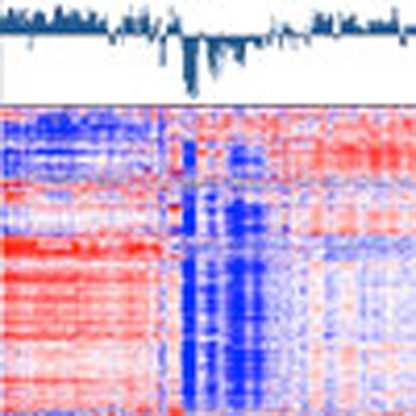

mRNA sequencing (RNA-Seq) provides digital profiling of gene expression with unprecedented depth, resolution and coverage^1^,^2^. To obtain reliable and reproducible RNA-Seq data, RNA quality is of paramount importance^3^. One challenge to obtaining high-quality RNA is RNA degradation before and during sample collection in preparation for RNA isolation due to tissue necrosis, which is an issue especially for samples collected in clinical settings and field studies^4^,^5^. For example, human transcriptome studies, such as the BrainSpan (http://www.brainspan.org) and Genotype-Tissue Expression (GTEx)^6^ projects, rely on post-mortem tissues. In addition, it is well documented that post-mortem intervals and cell stressors such as hypoxia can have substantial impact on RNA integrity^7^,^8^.

A widely adopted measure of RNA integrity is an electrophoretic-based method to derive an RNA integrity number (RIN)^9^,^10^. This method uses a machine-learning approach to extract features from electrophoretic traces (Bioanalyzer) and train a neural network that is predictive of RINs in a large set of samples manually curated by experts. In the context of RNA-Seq data analysis, several recent studies investigated how degraded RNA samples of different RINs can affect detection of differentially expressed genes^4^,^11^. By introducing experimentally controlled RNA degradation either before or after RNA extraction, these studies demonstrated that RINs reliably reflected the severity of degradation and that the use of degraded RNA samples could result in thousands of false positives, while the genuine changes could be masked if corrective measures were not implemented.

One apparent consequence of degraded RNA on RNA-Seq is the 3′ bias of read coverage along messenger RNA (mRNA) transcripts. Several quality control (QC) metrics have recently been developed to detect such bias, flag problematic samples and identify other technical issues^12^,^13^. However, to our knowledge, no systematic studies have carefully characterized RNA degradation in post-mortem samples and their impact on gene expression quantification with a wide range of tissue types across hundreds of samples. Such an analysis is urged to develop the optimal practices in mining the rich information provided by large-scale transcriptome sequencing projects and by meta-analysis of enormous data generated by individual studies available in public repositories^14^.

Several challenges are present in QC of RNA-Seq data derived from post-mortem tissues available in the public domain. First, there is currently no consensus with regard to the RIN required to generate high-quality data, and the criteria vary substantially depending on the specific studies used. Thus it is not unusual that re-analysis or meta-analysis of published data sets prefers more stringent filtering than the original studies that generated the data sets. Second, in public repositories, RINs of RNA samples used to generate RNA-Seq libraries are frequently not reported and thus re-analysis in follow-up studies has to perform QC on the RNA-Seq data directly. Third, the use of RINs as a measure of RNA-Seq data quality can have potential caveats. In particular, while a RIN is derived largely based on the integrity of ribosomal RNAs (rRNAs)^9^, the decay process of mRNA transcripts might be distinct and even gene specific, which is not necessarily reflected in the RIN completely.

To address these challenges, here we develop a method to estimate mRNA integrity number (mRIN) from RNA-Seq data sets directly based on quantitative modelling of the 3′ bias of read coverage. We applied this method to the large-scale RNA-Seq data sets generated by the BrainSpan and GTEx projects to demonstrate its effectiveness in quantifying mRNA degradation that markedly affected the global expression profiles. Unexpectedly, our method also suggests that the degree of degradation varies among different genes, and a substantial fraction of the variation can be explained by functional and structural features of the associated transcripts.

## Results

### 3′ bias in degraded RNA has a global impact on gene expression

The mechanism of RNA degradation in post-mortem samples during tissue necrosis is not well understood. The 3′ bias observed in RNA-Seq data could arise from RNA degradation by 5′ exonucleases^15^,^16^. Alternatively, RNA fragmentation by endonucleases or stochastic hydrolysis can also introduce the same apparent bias after poly-dT selection to purify polyadenylated mRNA, which is currently a standard step in mainstream RNA-Seq library preparation protocols including the Illumina TruSeq RNA Sample Prep Kit.

To demonstrate the importance of assessing mRNA integrity in RNA-Seq data derived from post-mortem tissue samples and to distinguish different types of mRNA degradation, we first used a large data set generated by the BrainSpan project. The dataset used in this analysis is composed of gene and exon quantifications of 578 samples derived from multiple brain structures of post-mortem human brains at different developmental stages (Supplementary Data 1). Importantly, Affymetrix HuEx exon microarrays were used as an independent platform to profile a largely overlapping set of samples (479 samples profiled by both platforms)^17^. While poly-dT selection was used to deplete rRNAs in RNA-Seq library preparation, it was not used to prepare complementary DNAs (cDNAs) for HuEx exon microarray hybridization^18^, a key difference of the two platforms. Therefore, partial mRNA fragmentation will least likely result in significant 3′ bias in HuEx probe signals along transcripts.

**Figure 1:**
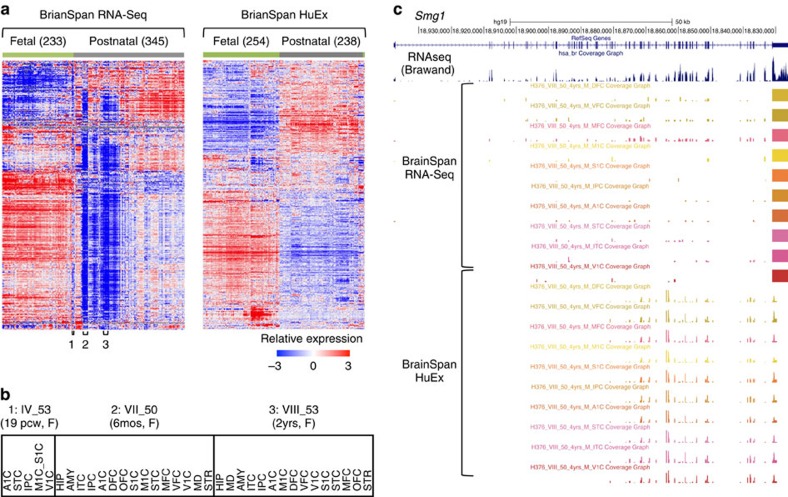
Impact of presumptive mRNA degradation on global gene expression profiling. (**a**) Gene expression profiles of post-mortem brain samples in the BrainSpan data as measured by RNA-Seq (left) and Affymetrix HuEx exon microarrays (right). Genes and samples are ordered by centroid linkage hierarchical clustering of each data set. In the RNA-Seq data, a subset of samples, frequently collected from the same individuals, show pervasive under-representation of gene expression (examples labelled with 1, 2 and 3 below the heat map). (**b**) Examples of fetal (1) and postnatal (2 and 3) brain samples derived from the same individuals for which the global gene expression profiles appear to be severely affected by RNA degradation. (**c**) *Smg1* as an example of discrepancy in RNA-Seq and exon array data with respect to 3′ bias. RNA-Seq and exon array data shown are from different brain structures of the same individual (VIII_50); 14/16 samples from this individual are ranked among the 50 most degraded samples. For comparison, a separate track shows brain RNA-Seq data derived from an independent study^47^. A1C, primary auditory cortex (core); AMY, amygdaloid complex; DFC, dorsolateral prefrontal cortex; HIP, hippocampus (hippocampal formation); IPC, posteroventral (inferior) parietal cortex; ITC, inferolateral temporal cortex (area TEv, area 20); M1C, primary motor cortex (area M1, area 4); MD, mediodorsal nucleus of thalamus; MFC, anterior (rostral) cingulate (medial prefrontal) cortex; OFC, orbital frontal cortex; S1C, primary somatosensory cortex (area S1, areas 3,1 and 2); STC, posterior (caudal) superior temporal cortex (area TAc); STR, striatum; V1C, primary visual cortex (striate cortex, area V1/17); VFC, ventrolateral prefrontal cortex.

We began with hierarchical clustering analysis of gene expression profiles^19^ as measured by RNA-Seq and microarrays, respectively, to obtain a global view of the developing brain transcriptome. For both data sets, clustering of samples almost perfectly separated fetal brains from neonatal and adult brains, which is consistent with the marked gene expression changes during development (Fig. 1a). However, there are important differences between the two data sets. In the RNA-Seq data set, a subset of samples show very distinct expression profiles characterized by low levels of expression in a vast majority—but not all—genes. Most of these samples are postnatal; some, however, are fetal. More careful examination shows that samples derived from the same individuals, regardless of brain regions or age, tightly clustered together (Fig. 1a,b). This vast, characteristic under-representation of gene expression was not observed in the same samples measured by microarrays (see also below).

One possible cause of the observed skew in the RNA-Seq data is that RNA-Seq is more sensitive to RNA quality than microarrays are (in particular, partial fragmentation of RNA), due to the poly-dT selection step. This can also explain why postnatal brains were affected more severely than fetal brains, because for the latter, high-quality tissues are more accessible.

To test this hypothesis, we first examined individual genes in samples with presumed RNA degradation. One example is the *Smg1* gene in samples derived from individual VIII_50, a 4-year-old male, which were profiled by both RNA-Seq and exon microarrays (Fig. 1c). While the microarray data show relatively uniform probe intensities from different exons throughout the transcript, the RNA-Seq data suffer from a severe 3′ bias and the reads are predominantly limited to the last exon. The low read coverage throughout the transcript, except the very 3′ end, has likely caused the underestimation of *Smg1* expression. These observations suggest that partial mRNA fragmentation is likely a major source of bias in gene expression quantification of degraded post-mortem brain samples.

### mRIN quantifies 3′ bias and alteration in gene expression

**Figure 2:**
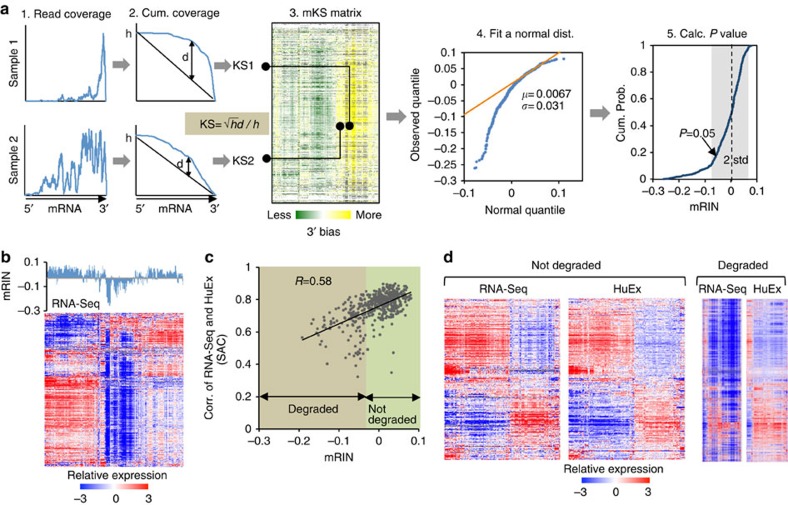
mRIN effectively measures mRNA integrity from RNA-Seq data. (**a**) Schematic illustration of the algorithm to estimate mRIN. After estimation of the 3′ bias of each gene and sample using a KS statistic from the read coverage profile, an mRIN is calculated for each sample. A normal distribution of the mRINs of non-degraded samples is estimated using a mixture model to assess the statistical significance. (**b**) Global under-representation of gene expression of the BrainSpan samples as measured by RNA-Seq is associated with low mRINs. Samples in the mRIN bar plot and the heat map are in the same order. (**c**) Validation of mRIN as a measure of mRNA integrity by a direct comparison of the RNA-Seq and exon array data. This analysis included 479 samples whose gene expression was quantified by both RNA-Seq and exon arrays. For each sample, the correlation of gene expression estimated from RNA-Seq and that estimated from exon arrays (denoted seq–array correlation or SAC) is calculated. SAC is plotted against the mRIN of each sample (Pearson correlation *R*=0.58, *P*<2.2 × 10^−16^, F-test). (**d**) mRIN was used to separate 124 samples with the most severe RNA degradation (mRIN<−0.033, *P*<0.1, Methods) from the remaining 355 samples. For each group, the heat maps of gene expression as measured by RNA-Seq and exon arrays are shown, with genes and samples in the same order as determined by hierarchical clustering of the array data.

To estimate mRNA degradation directly and quantitatively from RNA-Seq data, we went forward to develop a statistical method to quantify the 3′ bias of each gene, from which a single-number mRIN is derived for each sample (Fig. 2a; Methods). Specifically, for each gene and sample, mapped RNA-Seq reads are counted to obtain a read coverage profile across all exonic positions of a representative RefSeq transcript (step 1), which is converted into a cumulative coverage profile (step 2). This cumulative profile is then compared with a null distribution one would expect if reads are uniformly distributed. Change of the cumulative distribution due to 3′ bias is quantified by a modified Kolmogorov–Smirnov (KS) statistic^20^. To further mitigate additional biases that cannot be explained by the 3′ bias due to mRNA degradation (for example, sequencing artefacts due to local base compositions^21^, inaccuracy of transcript annotation or alternative RNA processing), we normalize the matrix of KS statistics by subtracting the median across samples (mKS matrix, step 3). Finally, the mRIN of each sample is defined as the negative of the average mKS values across all genes in the sample. With this measure, samples with negligible degradation are expected to have mRINs following approximately a normal distribution with a zero mean, while degraded samples have negative mRINs with larger deviations from which the statistical significance of degradation can be evaluated (steps 4 and 5; Supplementary Fig. 1).

In the analysis of the BrainSpan RNA-Seq data, a modification we adopted to obtain the mKS matrix and mRINs of samples is that read coverage profiles were estimated at the exon instead of the nucleotide resolution due to the availability of data (Fig. 1c); however, we expect this to have very moderate effects on mRIN (see below). As expected, the distribution of mRINs has a heavier tail on the left deviating from a normal distribution, indicating degraded RNA samples. In total, 141 (24%) and 170 (29%) samples were called to have significant degradation at *P*<0.05 and <0.1, respectively.

To validate the proposed mRIN as a measure of mRNA integrity, we first examined the mRINs together with the gene expression profiles of the associated samples. Indeed, samples with global under-representation of gene expression matched those with the most negative mRINs (Fig. 2b). To have a more direct and quantitative assessment, we compared gene expression profiles of the 479 samples that are profiled by both RNA-Seq and exon microarrays. We argued that the extent of degradation is reflected in the discrepancy between the RNA-Seq and microarray data, since the latter is not affected by partial RNA fragmentation. Therefore, for each sample, we calculated the correlation of the gene expression profiles as measured by the two platforms (that is, seq–array correlation), and examined how seq–array correlation relates to mRIN. The two measures are strongly correlated with each other (Pearson correlation *R*=0.58, *P*<2.2 × 10^−16^, F-test; Fig. 2c). We separated the samples into two groups depending on mRIN (using a conservative threshold: −0.033 or *P*=0.1), and examined the gene expression profiles of each group as measured by the two platforms (Fig. 2d). For the group of samples with no or minimal degradation, the gene expression profiles measured by the two different platforms are indeed very similar. In sharp contrast, the samples inferred to be severely affected by degradation have drastic difference in their expression profiles as measured by the two platforms, with vast under-representation in the RNA-Seq data. On the basis of these observations, we conclude that mRNA degradation due to partial fragmentation can have a marked impact on global expression profiles measured by RNA-Seq, and mRIN can be used to quantify such degradation.

### Comparison of mRIN with RIN and other QC metrics

**Figure 3:**
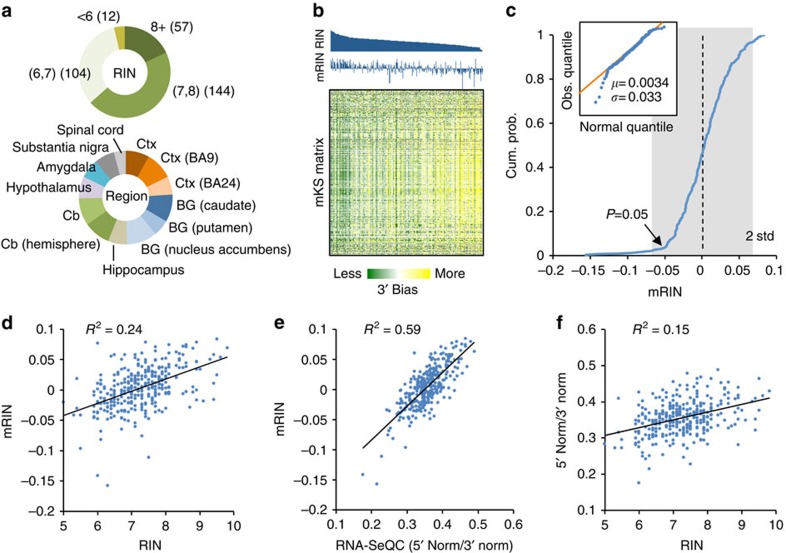
Comparison of mRIN with RIN and other QC metrics. (**a**) A summary of 317 GTEx brain samples with respect to RIN numbers and brain regions. BG, basal ganglia; Cb, cerebellum; Ctx, cortex. (**b**) The mKS matrix is shown with samples ordered by RINs and genes ordered by hierarchical clustering. Sample RINs and mRINs are shown at the top in the same order. (**c**) Cumulative distribution of mRINs. A heavier tail on the left side reflecting degraded samples is present, while the remaining samples fit well with a normal distribution as shown in the qqplot in the inset. *P* values are estimated from the estimated normal distribution. (**d**,**e**) Correlation between mRIN and RIN (**d**), between RNA-SeQC metric and mRIN (**e**) and between RNA-SeQC metric and RIN (**f**). Squared Pearson correlation is indicated in each scatter plot.

We next investigated how mRIN relates to RIN, which is currently a standard measure of RNA quality. Since matched RINs are currently not released publicly for RNA samples in the BrainSpan data set, we performed a second set of analyses on a large-scale human brain RNA-Seq data set obtained from the GTEx project^6^. This data set included 410 brain samples with matched RIN numbers. Alignment of the RNA-Seq data, QC and gene expression quantification with RNA-SeQC^12^ were performed by the GTEx project team^6^ and were directly used in our analysis. To minimize technical variations introduced in RNA-Seq library preparation and sequencing, we conservatively limited our analysis to 317 samples with a relatively high rate of read mapping (⩾67%). In this subset, 57 samples have an RIN ⩾8 (18%), 144 samples between 7 and 8 (45%) and 116 samples <7 (37%); these samples are from cortex, basal ganglia, cerebellum and other brain regions from multiple donors (Fig. 3a; Supplementary Data 2).

We estimated the mKS matrix and mRINs of the 317 samples from nucleotide-resolution read coverage profiles (Supplementary Data 2). When the samples were ordered by RINs, it is apparent from the mKS matrix that those with low RINs in general have stronger 3′ bias and thus lower mRINs, but this is not always the case (Fig. 3b). Similar to the BrainSpan data set, mRINs approximately follow a normal distribution, with a heavier tail on the left side indicating degraded RNA samples. By fitting this normal distribution, we estimated that 10 (3%) and 82 (26%) samples were degraded at *P*<0.05 and <0.25, respectively (Fig. 3c, Methods). We note the estimation of mRINs is extraordinarily robust with respect to the set of genes used for analysis, and genes randomly divided into two subsets gave essentially the same results (*R*^2^=0.9998, Supplementary Fig. 2).

When we compared RIN and mRIN quantitatively, 24% of variance in RINs across samples can be explained by their mRINs (*P*<2.2 × 10^−16^, F-test; Fig. 3d). Among the 57 samples with RIN ⩾8, the smallest *P* values are 0.16 and 0.17, while the remaining 55 samples (96%) have *P*>0.25. In contrast, among the 22 samples with RIN ≤6, only 6 (27%) have *P*>0.25. Therefore, the concordance of mRIN and RIN is remarkable, although the two methods estimate RNA integrity using very different approaches with different underlying assumptions.

Several other tools have been developed recently to gauge 3′ bias and flag-degraded RNA samples^12^,^13^, although systematic evaluation of their performance in large-scale data sets has not been reported. Among these, RNA-SeQC calculates the read coverage at the 5′ or 3′ end of each transcript (for example, the first or last 50 nucleotides used in the GTEx data set) normalized by the coverage throughout the transcript. These quantities are averaged across all genes, denoted 5′ Norm and 3′ Norm, to evaluate the overall 3′ bias of the sample. We found a good correlation between mRIN and the 5′ Norm/3′ Norm ratio (*R*^2^=0.59, Fig. 3e), and, to a lesser extent, between mRIN and each of the two metrics (Supplementary Fig. 3a,b). This is not surprising because both mRIN and RNA-SeQC quantify 3′ bias. However, the 5′ Norm/3′ Norm ratio by RNA-SeQC explains only 15% of variance in RINs and even less if the two metrics were used separately (Fig. 3f; Supplementary Fig. 3c,d). Furthermore, no correlation between RIN and the 5′ Norm/3′ Norm ratio remains after mRIN is controlled (*R*^2^=0.0001, *P*=0.84, F-test; Supplementary Fig. 3e). The better concordance of mRIN with RIN suggests the advantage of mRIN over RNA-SeQC in quantifying 3′ bias with a carefully designed statistical model.

As we argued earlier, RIN provides a very robust measure of RNA integrity but does not necessarily capture all aspects of mRNA degradation. This is in line with the imperfect correlation of RIN and mRIN, although the comparative advantage of the two metrics has to be assessed by an independent measure, for example, by evaluating how each method detects alterations in gene expression profiles resulting from RNA degradation. Since independent expression quantifications immune to 3′ bias are not available for the GTEx data set, we sought an alternative strategy. Specifically, we argue that samples with high RIN and mRIN (RIN⩾8 and mRIN⩾0, respectively) have minimal degradation and can be used as a surrogate of the ‘reference transcriptome’. The expression correlation of the remaining samples with the reference samples is expected to decrease depending on the severity of degradation.

**Figure 4:**
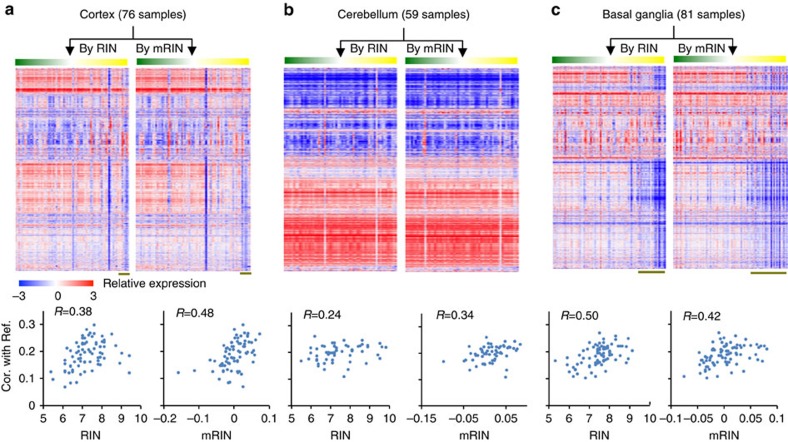
Comparison of mRIN and RIN in predicting altered gene expression profiles. (**a**) Top panel: gene expression profile of GTEx cortical samples ordered by RIN and mRIN, respectively. Bottom: scatter plot showing RIN (left) and mRIN (right) of each sample (*x* axis) and their median correlation of expression with a subset of high-quality reference samples (*y* axis). (**b**,**c**) Similar to **a**, but for samples from cerebellum (**b**) and basal ganglia (**c**). Note that log2 expression values are median-centered to highlight the contribution of genes with variable levels among samples to the correlation metrics.

To minimize the heterogeneity of gene expression among different brain regions, we analysed samples from the cortex, cerebellum and basal ganglia separately, as these regions display distinct expression profiles (Supplementary Fig. 4). When we ordered samples by RIN or mRIN, it became clear that a subset of cortical and basal ganglia samples with low RIN or mRIN have distinct gene expression profiles compared with high-quality samples in these regions, although this is less apparent for the cerebellum (Fig. 4a–c, top panels). Quantitatively, both RIN and mRIN effectively predicted the correlation of each sample with the high-quality reference (Fig. 4a–c, bottom panels, Pearson correlation *R* between 0.24 and 0.50 for RIN and 0.34 and 0.48 for mRIN). Importantly, mRIN is more predictive than RIN in the cortex and cerebellum. Therefore, mRIN is very comparable to RIN in isolating degraded samples with altered expression profiles, although the two methods appear to capture both shared and distinct aspects of mRNA degradation, which can be explained in part by the source of degradation modelled by each method.

### Gene-specific degradation and distinct transcript features

**Figure 5:**
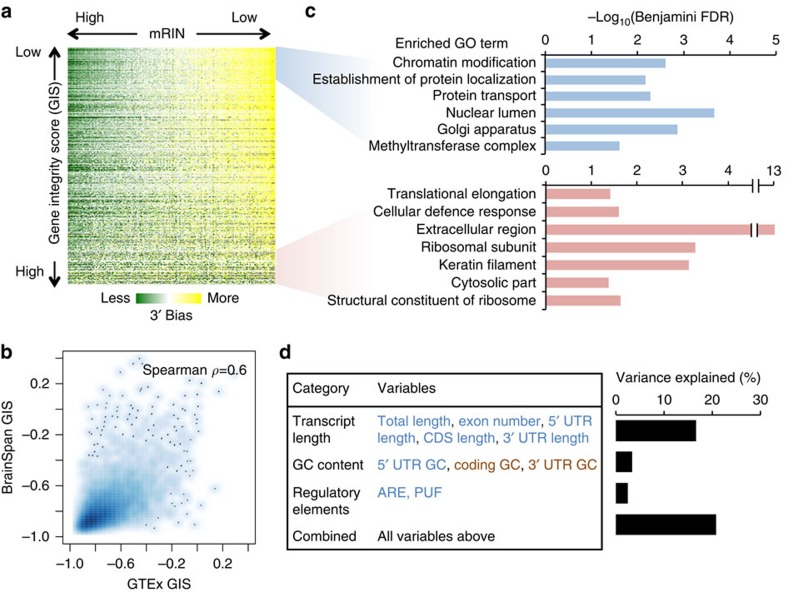
Gene-specific degradation and their functional and transcript structural features. (**a**) Visualization of the mKS matrix with samples ordered by mRIN and genes ordered by a gene instability score (GIS) defined as the correlation between mRIN and mKS across samples. (**b**) Smooth scatter plot of GIS estimated from the GTEx and BrainSpan data sets, respectively. Areas in darker blue represent a higher density of genes. (**c**) Gene ontology (GO) analysis of genes with the lowest or highest GIS. Only representative GO terms with Benjamini FDR <0.05 are shown (see Supplementary Data 4 and 5 for a complete list). (**d**) Linear regression analysis of GIS using different groups of features reflecting transcript length, base composition and regulatory sequences. Individual variables showing positive or negative correlation with transcript stability are indicated in red and blue, respectively. Variance explained by each regression model including a specific subset of variables is shown in the bar plot.

Besides a per-sample summary of mRNA integrity by mRIN, our method has the advantage of being able to evaluate gene-specific degradation. In both the BrainSpan and GTEx data sets, not all genes appear to be affected by 3′ bias to the same degree, as one can tell from the expression profiles (Fig. 2b,d; Fig. 4a–c), but more directly, from the mKS matrices (Figs 2a and 5a).

To investigate the mechanism of gene-specific degradation, we calculated a gene integrity score (GIS) by correlating mKS values of each gene and the associated mRINs across all samples (Supplementary Data 3). We found that GIS values estimated from the GTEx data set and the BrainSpan data set are highly correlated (Spearman *ρ*=0.6, *P*<2.2 × 10^−16^, F-test; Fig. 5b). This is remarkable, given that the mKS matrices of the two data sets were calculated from read coverage profiles at different resolutions (per-exon for BrainSpan and per-nucleotide for GTEx). Not surprisingly, the use of RIN instead of mRIN to calculate GIS resulted in quantitatively similar scores (Supplementary Fig. 5), as one would expect from the correlation between mRIN and RIN. In addition, most genes have negative GIS, suggesting more severe 3′ bias in more degraded samples in general, although the degree varies. Our analysis thus focused on GIS estimated from the GTEx data set.

We asked whether there is any functional bias of genes associated with transcript stability in post-mortem samples. Since the GIS scores show a continuum without obvious cutoffs to separate different groups of genes, we examined the top and bottom 1,000 genes and their gene ontologies^22^. The top 1,000 genes with the most unstable transcripts show significant enrichment in those involved in gene expression regulation, such as chromatin modification (Benjamini false discovery rate (FDR) <0.002; Fig. 5c). Genes involved in mRNA processing also appear to be enriched, although somewhat moderately (Benjamini FDR=0.18; Supplementary Data 4). On the other hand, the bottom 1,000 genes with the most stable transcripts are enriched in ribosome subunits and extracellular proteins (Benjamini FDR <5 × 10^−4^ and <5 × 10^−14^ , respectively; Fig. 5c; Supplementary Data 5). These observations suggest that the degradation process is not completely random.

Encouraged by this finding, we also investigated what transcript features can predict their stability as reflected in GIS. We found longer transcripts are more unstable and the transcript length alone explains about 15% of the variance in GIS (*P*<2.2 × 10^−16^, F-test); inclusion of 3′ untranslated region (UTR), coding sequence (CDS) and 5′ UTR sizes, as well as exon numbers together with the transcript length in a linear regression model only moderately increased the explained variance (16.6%, *P*<2.2 × 10^−16^, F-test; Fig. 5d; Supplementary Fig. 6). We also examined base composition (GC content) in different regions of the transcripts, as well as AU-rich elements (AREs) and PUM2 motif sites in 3′ UTRs, which are reported to affect mRNA stability^23^,^24^,^25^,^26^. Base composition and regulatory sequences each explain 3.4 and 2.4% of the variance in GIS (*P*<2.2 × 10^−16^, F-test). Interestingly, while unstable transcripts tend to have a higher AU-content in 3′ UTR and CDS, they tend to have a higher GC content in the 5′ UTR (Supplementary Fig. 7). Both ARE and PUM2 motif sites are associated with unstable transcripts, even after normalization of 3′ UTR length (Supplementary Fig. 8). When all these variables were included in multiple regression analysis, a total of 20.8% of the variance in GIS can be explained (Fig. 5d). Therefore, while these features are insufficient to predict the stability of individual transcripts, they are predictive for the population average among groups of transcripts that share these features.

## Discussion

For human transcriptome studies that rely on post-mortem tissues, the first and probably the most challenging issue is RNA degradation during sample collection due to the occurrence of tissue necrosis, so that partially degraded samples have to be used in certain scenarios. Similar technical challenges are likely present in the collection of samples that require prolonged processing prior to RNA extraction (for example, laser capture microdissection). In this study, we developed a statistical method to evaluate mRNA integrity directly from RNA-Seq data by quantitatively modelling the 3′ bias of read coverage. Application of this method to two independent, large-scale RNA-Seq data sets profiling human post-mortem tissues demonstrated that degraded RNAs can result in strong 3′ bias that markedly skews global gene expression profiles with standard pipelines of data processing. Such bias may mask genuine biological differences such as brain region-specific expression or expression variation among different individuals, if it is not properly controlled. To our knowledge, this is the first systematic characterization of mRNA degradation in RNA-Seq data derived from post-mortem samples on such a large scale.

Our analysis warrants additional caution in processing data from samples affected by degradation. Whether such samples should be excluded for analysis largely depends on specific studies and the extent to which the resulting bias can be minimized by computational normalization methods. In particular, the decision can differ between the original studies generating the data sets and follow-up studies that perform re-analysis or meta-analysis of published data. Unfortunately, the quality of RNA samples used for RNA-Seq could be somewhat obscure for follow-up analysis of published data, and sample RINs are sometimes not reported. Our intention was thus to develop a computational method to provide *post hoc* assessment of mRNA quality directly from RNA-Seq data independent of RIN or other prior knowledge in the context of meta-analysis. We demonstrated the effectiveness of our method in comparison with RIN and other existing QC metrics in isolating degraded samples. It also has to be emphasized that a number of approaches and software tools have recently been developed to normalize biases in RNA-Seq data introduced by mRNA degradation and other technical issues^12^,^13^,^27^,^28^,^29^,^30^,^31^. Therefore, mRIN can be potentially incorporated into some of these methods to address these issues.

In living cells, control of mRNA stability is critical for both mRNA surveillance and post-transcriptional gene expression regulation. The molecular mechanisms underlying such controls are complex and only partially understood^15^,^16^. RNA degradation pathways involve a number of RNases, including endonucleases that cut inside the transcripts and 5′ and 3′ exonucleases that digest RNA from the two termini. These RNases are assembled into different multiprotein complexes coupled with other steps of RNA processing such as decapping and deadenylation^15^,^32^,^33^. Their substrate specificity is conferred by many co-factors and specific regulatory sequences embedded in the target transcripts.

Through comparison of the expression profiles as measured by RNA-Seq and exon microarrays, our analysis suggests that partial fragmentation of mRNA is a major source of bias in RNA-Seq data derived from post-mortem tissue samples. How such fragments are generated and accumulated during tissue necrosis is not clear, but the process might be a combined consequence of several possible mechanisms^4^. For example, active endonucleolytic cleavage can occur in specific transcripts when the cellular mRNA decay machineries initially remain active, which can be followed by leakage of extracellular RNases (such as RNase A) into the cells and stochastic RNA hydrolysis in the deteriorating cellular environment.

An unexpected finding of this study is that there seems to be a reproducible and gene-specific component of mRNA degradation in post-mortem samples, as recently noted in another tissue type^4^. Intriguingly, our analysis revealed that transcript stability is associated with distinct functional groups and structural features, and that these observations agree well with previous cell-based studies of mRNA half-lives^34^,^35^,^36^. For example, genes playing regulatory functions, including chromatin-modifying enzymes, have been found to be unstable at both transcript and protein levels, while transcripts encoding housekeeping genes, such as the ribosome and extracellular proteins, are quite stable. Transcript length and regulatory sequences previously found to be associated with stability are also predictive in our analysis. These observations imply that mRNA degradation in post-mortem samples might be reminiscent of the active decay process in living cells depending on intrinsic properties encoded in the transcripts so that the high-level organization of the transcriptome can be recapitulated in our analysis. Further support of this ‘active degradation’ model comes from several recent studies demonstrating that endonucleolytic cleavage in the mammalian transcriptome, including the brain, is more widespread than previously appreciated^37^,^38^,^39^. This finding is particularly exciting because of technical challenges in global measurements of mRNA turnover. Specifically, current methods rely on pulse labelling of nucleosides or inhibition of transcription^35^,^40^,^41^ to uncouple mRNA synthesis and degradation^34^,^39^,^40^, which have essentially limited their application in cell cultures. We are therefore tempted to propose GIS as an alternative to evaluate mRNA stability in native tissues, although this strategy has to be evaluated more extensively in future studies.

## Methods

### The statistical model to quantify mRNA integrity

mRIN provides a single-parameter measurement of mRNA degradation, or more specifically partial mRNA fragmentation, for each sample. This method is based on the global 3′ bias of RNA-Seq read coverage in libraries prepared from degraded RNAs using the standard protocols, including poly-dT selection. In this study, we used RefSeq transcripts as gene models to quantify 3′ bias; for genes with multiple RefSeq transcripts, the longest transcript was used as a representative. To avoid ambiguity in read assignment to genes, only those genes without an overlap with other neighbouring genes were included in this study.

After mapping RNA-Seq reads to the reference genome and exon junctions, the mapped reads are counted in each exonic nucleotide to obtain the read coverage profile along mRNA transcripts. The coverage profile is then transformed into a cumulative distribution at nucleotide resolution. Denote the number of reads at each exonic position *x* (starting from the 3′ end of each transcript) as *n*_*x*_. The cumulative read coverage is denoted as




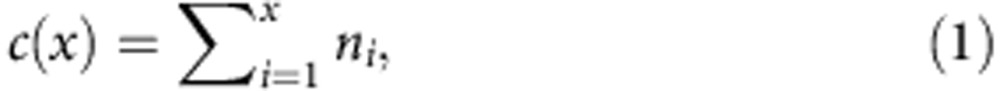




and the total nucleotide coverage is thus




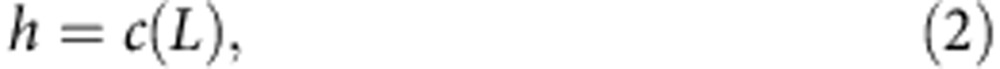




where *L* is the transcript length.

When there is no degradation of RNA or technical bias during library preparation and sequencing, RNA-Seq reads are expected to be uniformly distributed (the null distribution). In practice, certain amount of non-uniformity is always present, and a major source of such non-uniformity is the 3′ bias due to RNA degradation. Such deviation from the null distribution can be quantified by a modified KS statistic^20^:




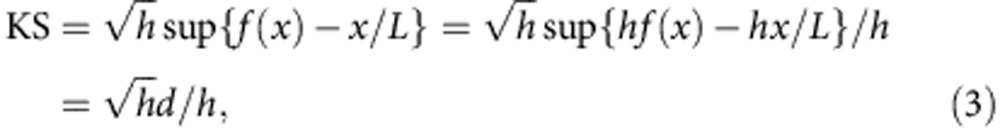




where *f*(*x*) is the cumulative read coverage distribution at position *x*, *f*(*x*)=*c*(*x*)/*h*.

Note that the direction of deviation is distinguished in *d*, so that the KS statistic is positive in the presence of 3′ bias and negative in the presence of 5′ bias.

To account for additional gene-specific bias (such as local base composition or errors in transcript annotation) that cannot be explained by the 3′ bias, the KS statistics are median-centred across all samples for each gene to obtain mKS values.

The mRIN of each sample is defined as the negative of average mKS statistics of all *N* genes:




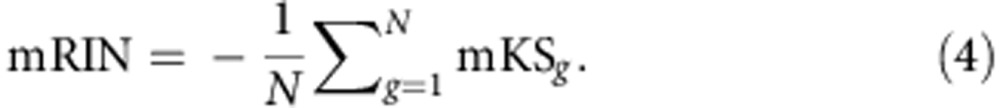




The negation is used so that degraded samples with 3′ bias have smaller mRINs analogous to RIN.

We note that mRINs of non-degraded samples should follow a normal distribution with approximately a zero mean *μ*=0. This is based on the assumption that the mKS statistics of different genes in a sample without degradation are independently and identically distributed. mRIN of the sample, which is the negative average of the mKS statistics across a large number of genes, is expected to converge to a normal distribution based on the central limit theorem. On the other hand, degraded RNA samples are expected to have more negative mRINs, resulting in a heavier tail on the left side of the distribution.

We can estimate the s.d. of mRINs of the null distribution (no degradation), denoted as *σ*, from samples with positive mRINs (which are least degraded) by the scaled median absolute deviation. For each sample, a *z*-score and *P* value can then be derived to estimate the probability of observing a specific mRIN under the null hypothesis (using a single-sided test):




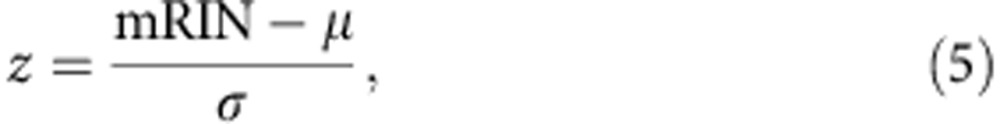







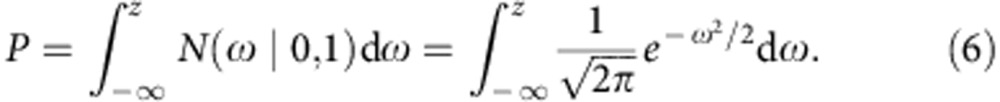




where *ϕ*(*ω*)=*N*(*ω*|0,1) is the probability density function of the standard normal distribution.

We also consider a scenario in which a relatively large number of samples in the data set might be degraded, such that the mean mRIN of non-degraded samples is shifted from zero. To address this concern, we sought to develop a more robust approach to estimating the null distribution. In brief, we assume samples with mRINs above a certain cutoff are non-degraded and these samples follow a truncated normal distribution, whose parameters can be estimated analytically.

For simplicity, suppose we already know the cutoff of the mRIN *α* and samples with mRIN>*α* are regarded as non-degraded samples. mRINs of the non-degraded samples follow a truncated normal distribution,




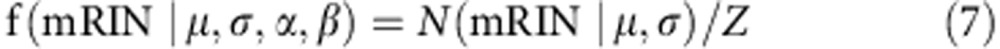




where 
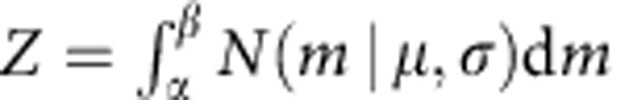
 and *β*=+∞. The mean and variance of the truncated normal distribution are^42^




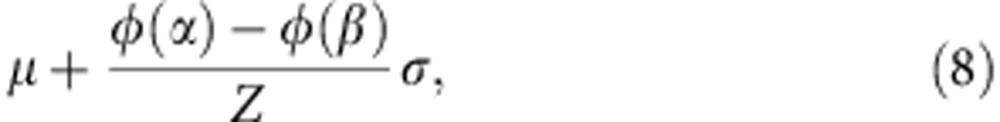




and




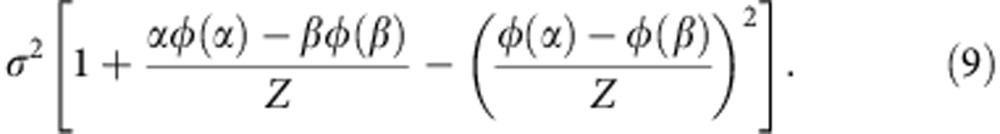




The parameters *μ* and *σ* can be derived from the moment estimator by solving the following equation system,




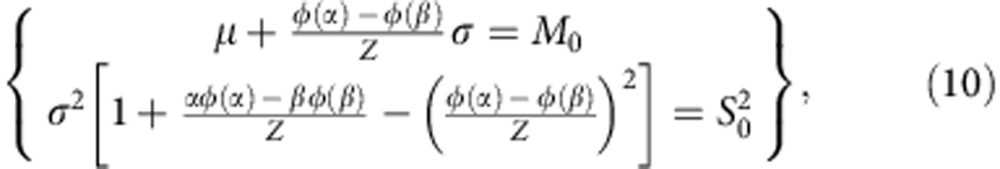




where *M*_0_ and 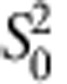 are the estimated mean and variance of the mRINs of the (truncated) non-degraded samples, respectively.

Since we do not actually know *α*, we perform a step search of the optimal *α* from −0.05 to 0.05, stepped by 0.0005 and calculate the estimated parameter *μ* and *σ*. For each *α*, the goodness of fit is evaluated by comparing the cumulative distribution of truncated mRINs and samples from the fit normal distribution using a KS statistic *D* (Supplementary Fig. 1). The threshold *α* that gives the smallest *D* is chosen. The estimated normal distribution is then used to calculate the *z*-score and *P* value of each sample.

### BrainSpan RNA-Seq and Affymetrix HuEx exon array data

For this study, we used an RNA-Seq data set of developing brain transcriptomes generated as part of the BrainSpan project (http://www.brainspan.org, accessed in March 2013). This was the latest version available at the time of analysis, but an updated version as since become available at the time of writing. This data set is composed of a large panel of post-mortem human brain samples that span 13 developmental stages including fetal, neonatal and adult brains. For each donor, samples were collected from 8 to 16 brain structures. A total of 578 samples were profiled by RNA-Seq using poly-dT-selected mRNAs. In parallel, the BrainSpan project also used Affymetrix HuEx ST exon microarrays to profile gene expression on a largely overlapping set of samples. In total, the exon array data set is composed of 492 samples, among which 479 have matched RNA-Seq data. Poly-dT selection was not used in preparation of cDNAs for exon array hybridization; instead, cDNAs were generated by random priming, because rRNAs are not expected to hybridize with probes designed for mRNAs.

The RNA-Seq data were available publicly as read per kilobase per million (RPKM) values at the exon and gene level (GENCODE V3C). These values were derived from read mapping using ELAND2, followed by quantification using RSEQtools^43^. The exon array data were available as exon and gene intensities normalized by the RMA algorithm implemented in the Affymetrix Power Tools. More details of data processing as performed by the BrainSpan project team were described in their documentation available at http://www.brainspan.org/static/help. These processed files were downloaded from http://www.brainspan.org/static/download.html.

To perform mRIN analysis, we used read coverage profiles derived from exon RPKM values, because the nucleotide-resolution read coverage profiles or the raw data were not available. In this case, the total read coverage *h* (average RPKM × *L*) reflects the abundance and length of each transcript, but not the sequencing depth of each sample. However, assuming the sequencing depth is relatively uniform across different samples, we do not expect this to have significant impact on the results. To reduce uncertainty, we estimated the KS statistic only for genes with RPKM >2 in each sample and assigned a missing value otherwise. After we obtained the mKS matrix, 7,783 genes with mKS estimated in >50% of samples were used to calculate mRINs. For this study, we used the mixture model to estimate the parameters of the null distribution (*α*=0.003, *μ*=0.0066 and *σ*=0.031).

Centroid linkage hierarchical clustering of gene expression profiles presented in Fig. 1 used 9,075 of 18,979 genes in the RNA-Seq data (RPKM>3 in ⩾10 samples, log_2_-transformation, s.d.>1 and then median centred) and 6,550 of 17,604 genes in the microarray data (log_2_ intensity >7 in ⩾10 samples, s.d.>0.6 and median centred).

To determine the order of samples and genes described in Fig. 2d, we focused on 6,550 genes that passed filtering in exon array data. Among these, 6,319 were common in the RNA-Seq data and were used for a direct comparison. A hierarchical clustering was performed on these common genes across the 479 common samples using the exon array data. The samples were then divided into two groups with a threshold of mRIN=−0.33 (*P*=0.1). In each group, a heat map was generated with the predetermined order using each data set.

### GTEx RNA-Seq data

The current release of the GTEx data set (phe000006.v1, release date: 17 January 2014) included a total of 410 brain samples with RNA-Seq data, matched RINs, gene expression quantification and RNA-SeQC results, which were obtained from the GTEx project through dbGaP (https://dbgap.ncbi.nlm.nih.gov). Among these, 317 samples with a read mapping rate ⩾0.67 (based on RNA-SeQC results) were used in our analysis, because our initial examination of the gene expression profiles suggested that samples with a low mapping rate have lower correlations with the other samples. There appear to be a few additional ‘outlier’ samples as judged from their expression profiles (Fig. 4), but we did not find apparent abnormalities in QC metrics, so they were not excluded.

Alignment of RNA-Seq reads (by TopHat^44^ as provided by the GTEx project team) were extracted from bam files using bedtools^45^, which were converted into read coverage profiles along exonic positions of representative RefSeq transcripts.

We estimated KS statistics for each gene and sample with average read coverage *h*/*L*⩾2 and RPKM⩾2 (and missing value otherwise). To reduce potential noise in estimating mRIN, we applied filters of genes based on KS statistic and gene expression RPKM values calculated by RNA-SeQC. We first excluded the top 5% of genes with the smallest s.d. in their KS statistics across samples. To remove genes that show strong 5′ bias, typically due to errors in transcript annotation, alternative RNA processing or other technical issues, we excluded genes with a median KS statistic across samples <0. Finally, 9,168 genes with KS estimated in >50% samples were kept to derive the mKS matrix and calculate mRIN for each sample. The parameters of the null normal distribution were estimated to be *α*=0.0035, *μ*=0.0034 and *σ*=0.033.

To evaluate gene-specific integrity, we focused on 8,493 of 9,168 filtered genes above that are protein-coding genes with annotated 5′ UTR, CDS (whose length is multiple of three) and 3′ UTR. GIS is defined as the Pearson correlation between mKS values across all samples and their associated mRINs.

To correlate GIS estimated from the GTEx and BrainSpan data sets, we considered only genes with ⩾5 exons, because for the BrainSpan data set the read coverage profile is available only at the per-exon level, hence it is more difficult to obtain a precise estimate of KS values and thus GIS for genes with single or few exons.

To calculate the correlation of gene expression profiles across samples, as presented in Fig. 4, we required genes to have RPKM >3 in ⩾10 samples to eliminate low-abundance genes. RPKM values of 11,958 filtered genes are log_2_-transformed and median centred to calculate correlations of each sample with a subset of high-quality reference samples (defined by RIN⩾8 and mRIN⩾0). For each sample, the median correlation with the reference was then calculated. For a sample belonging to the reference, the median was calculated using the remaining samples in the reference set. To generate the gene expression heat maps shown in Fig. 4, we further required s.d. across all samples >0.6 and %presence ⩾0.8 to highlight genes with the most variations among samples. A total of 7,000 filtered genes were included for centroid linkage clustering analysis.

### Transcript structural and functional features

Representative RefSeq transcripts used to estimate mRIN were used to define the number of exons, whole-transcript length as well as the lengths of 5′ UTR, CDS and 3′ UTR. Exonic sequences of each of these regions were extracted to calculate GC content and search for motif sites. For AREs, we initially searched different variations (for example, AUUUA, WWAUUUAWW and others)^46^, but found AUUUA as the only one with significant correlation with GIS. We therefore focus on this core motif in our analysis. For the PUM2 motif, we used the consensus UGUAHAUA, as determined by previous studies^26^.

To relate transcript stability with transcript features, we first generated bins of 100 genes and calculated the averages of each feature in bins and their correlation with the average GIS in respective bins. For regression analysis, we first applied Fisher transformation on GIS, ln[(1+GIS)/(1−GIS)]/2, so that it follows approximately a normal distribution. For the same reason, we also applied log transformation on length of whole transcripts, as well as lengths of 5′ UTR, CDS and 3′UTR. Linear regression analyses were first performed using individual groups of variables and then in combination, as summarized in Fig. 5d.

Gene ontology analysis was performed using the online tool DAVID^22^. All genes included for GIS analysis were used as background to compare with the 1,000 most unstable and the 1,000 most stable transcripts, respectively.

### Software implementation

A set of perl and R scripts were implemented to calculate the exonic read coverage, cumulative distribution and KS statistics, mRINs and the statistical significance. The software package and the documentation are available through http://zhanglab.c2b2.columbia.edu/index.php/mRIN. Hierarchical clustering was performed using cluster (http://bonsai.hgc.jp/~mdehoon/software/cluster/) and java treeview (http://jtreeview.sourceforge.net).

## Additional information

**How to cite this article**: Feng, H. *et al*. mRIN for direct assessment of genome-wide and gene-specific mRNA integrity from large-scale RNA-sequencing data. *Nat. Commun.* 6:7816 doi: 10.1038/ncomms8816 (2015).

## Supplementary Material

Supplementary Figures Supplementary Figures 1-8

Supplementary Data 1 List of samples and the associated mRINs in the BrainSpan dataset.

Supplementary Data 2 List of brain samples and the associated mRINs in the GTEx dataset.

Supplementary Data 3 List of genes, their transcript features and GIS.

Supplementary Data 4 Gene Ontology terms associated with the most unstable transcripts. Marginally significant terms (Benjamini FDR>0.05) are shown in gray.

Supplementary Data 5 Gene Ontology terms associated with the most stable transcripts. Marginally significant terms (Benjamini FDR>0.05) are shown in gray.
