# Supplementary material for: mRIN for direct assessment of genome-wide and gene-specific mRNA integrity from large-scale RNA-sequencing data
Source: Nat Commun. 2015 Aug 3;6:7816. doi: 10.1038/ncomms8816 (PMC4523900; doi:10.1038/ncomms8816)
Supplement: Supplementary Figures — 1-8 [file ncomms8816-s1.pdf]

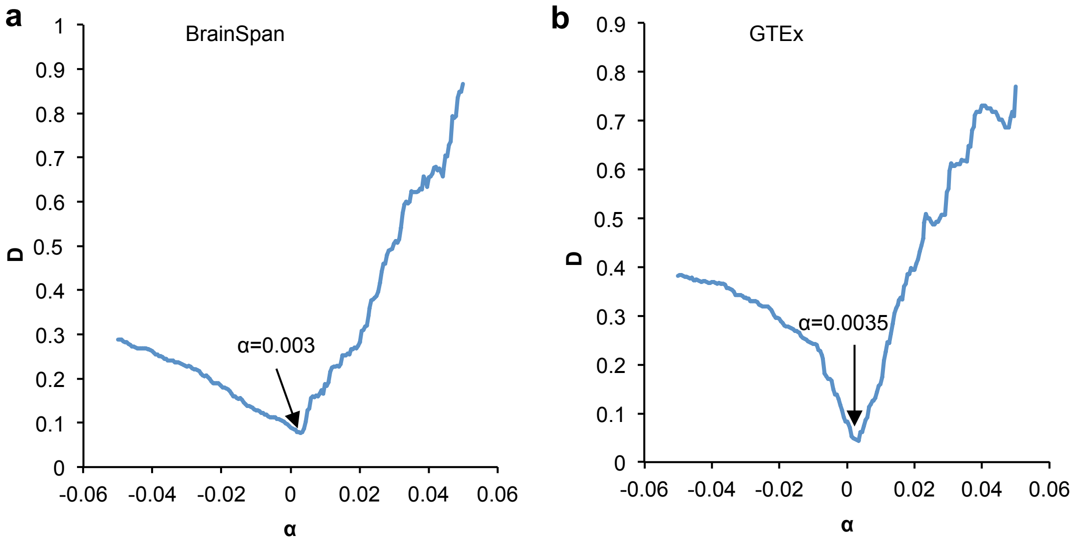

Supplementary Figure 1: Goodness of fit of mRIN to the normal distribution. The KS statistic (i.e., the maximum distance *D* between the empirical cumulative distribution of mRINs and the fit normal distribution) is plotted against the parameter *α***.**

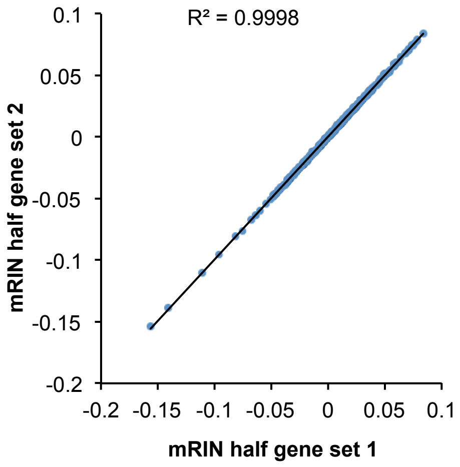

Supplementary Figure 2: Correlation of mRIN estimated from independent sets of genes using the GTEx dataset.
Genes were randomly divided into two halves of equal size, and for each sample, an mRIN was estimated from each subset of genes. The correlation of the two independent estimates is indicated.

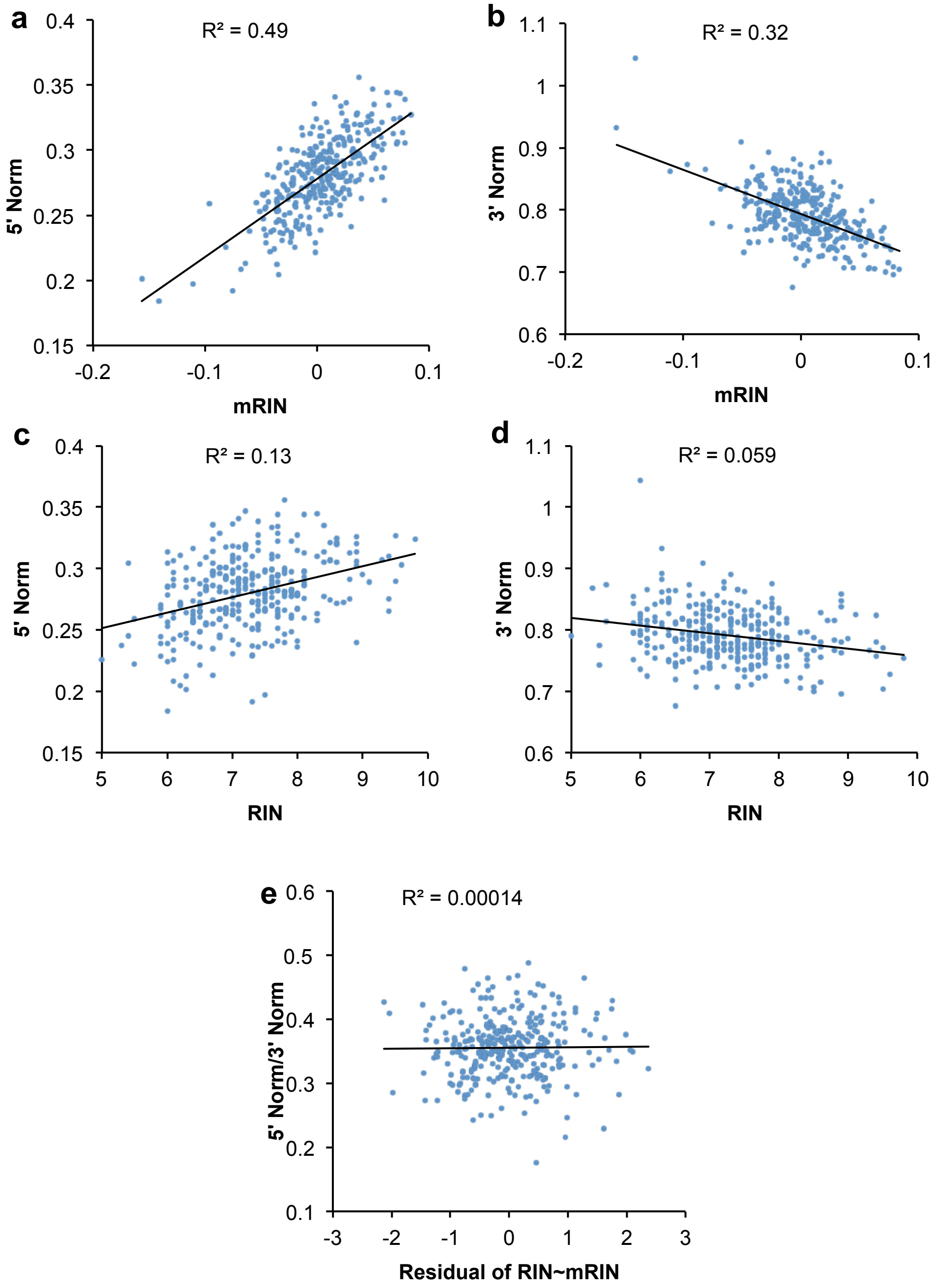

Supplementary Figure 3: Correlation of RNA-SeQC metrics with RIN or mRIN.
a,**b**, 5´ Norm (**a**) and 3´ Norm (**b**) with mRIN. **c**,**d**, 5´ Norm (**c**) and 3´ Norm (**d**) with RIN. **e**, The 5´ Norm/3´ Norm ratio and the residual of RIN predicted from mRIN.
Squared Pearson correlation is indicated in each panel. Note the ratio of the two RNA-SeQC metrics shows a higher correlation with mRIN or RIN (**Fig. 3e** and **f** in the main text).

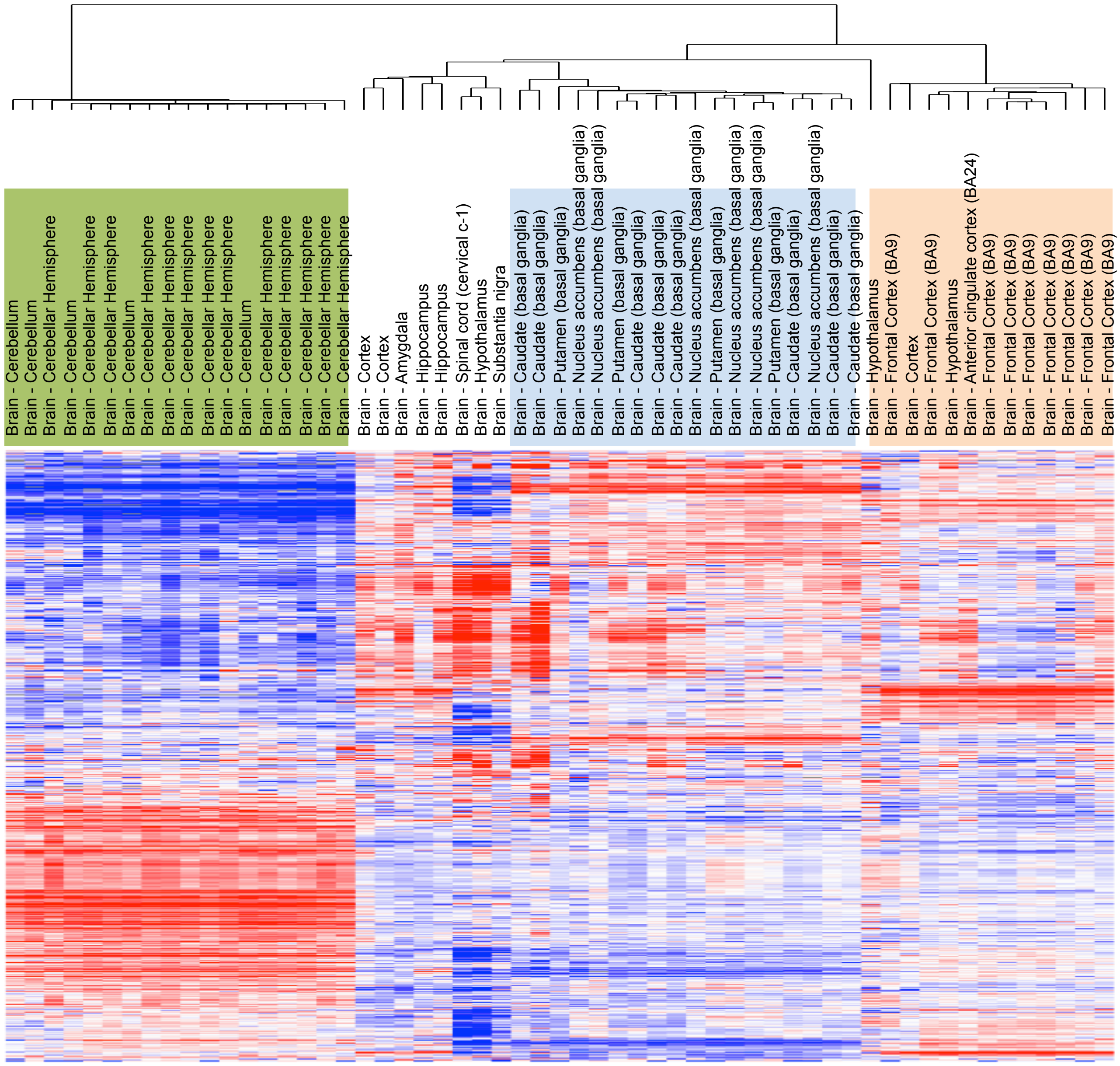

Supplementary Figure 4: Hierarchical clustering of GTEx brain samples based on gene expression profiles.

Only samples with RIN≥8 were included in this analysis. Note the three major clusters of samples from cerebellum, basal ganglia, and cortex, respectively (shaded).

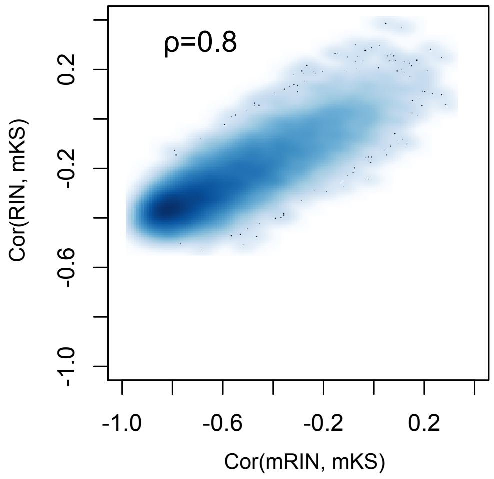

Supplementary Figure 5: GIS estimated from mRIN (x-axis) or RIN (y-axis) used to correlate with the mKS values of each gene.
A smooth scatterplot is shown with denser regions in darker blue.

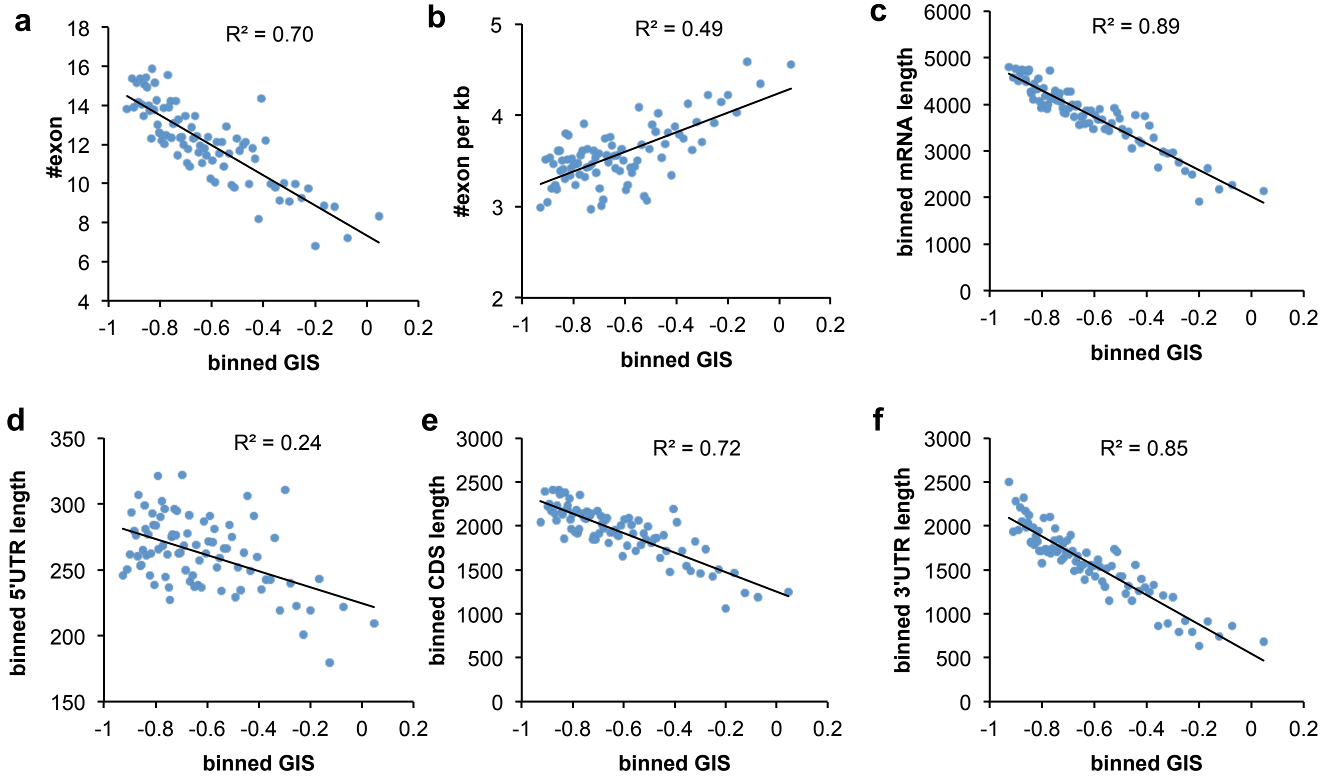

Supplementary Figure 6: Correlation of GIS with transcript length and structure. **a**, Number of exons. **b**, Number of exons normalized by transcript length. **c-f**, Lengths of the whole transcript (**c**), 5´ UTR (**d**), CDS (**e**), and 3´ UTR (**f**). The averages of the respective variables in bins of 100 genes are plotted against the average GIS in each bin.

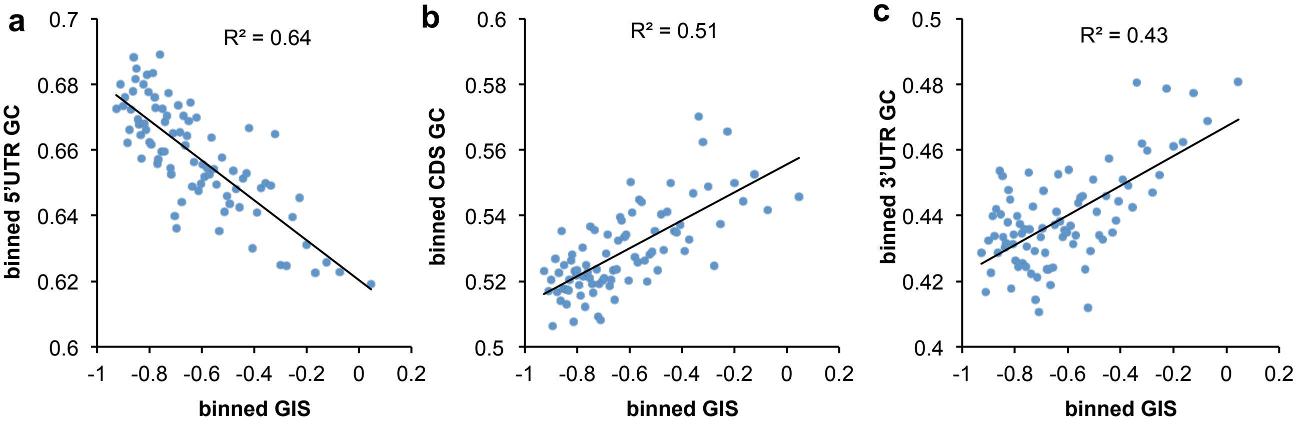

Supplementary Figure 7: Correlation of GIS with GC content in different regions of transcript.

a, 5´ UTR. **b**, CDS. **c**, 3´ UTR. The averages of the respective variables in bins of 100 genes are plotted against the average GIS in each bin.

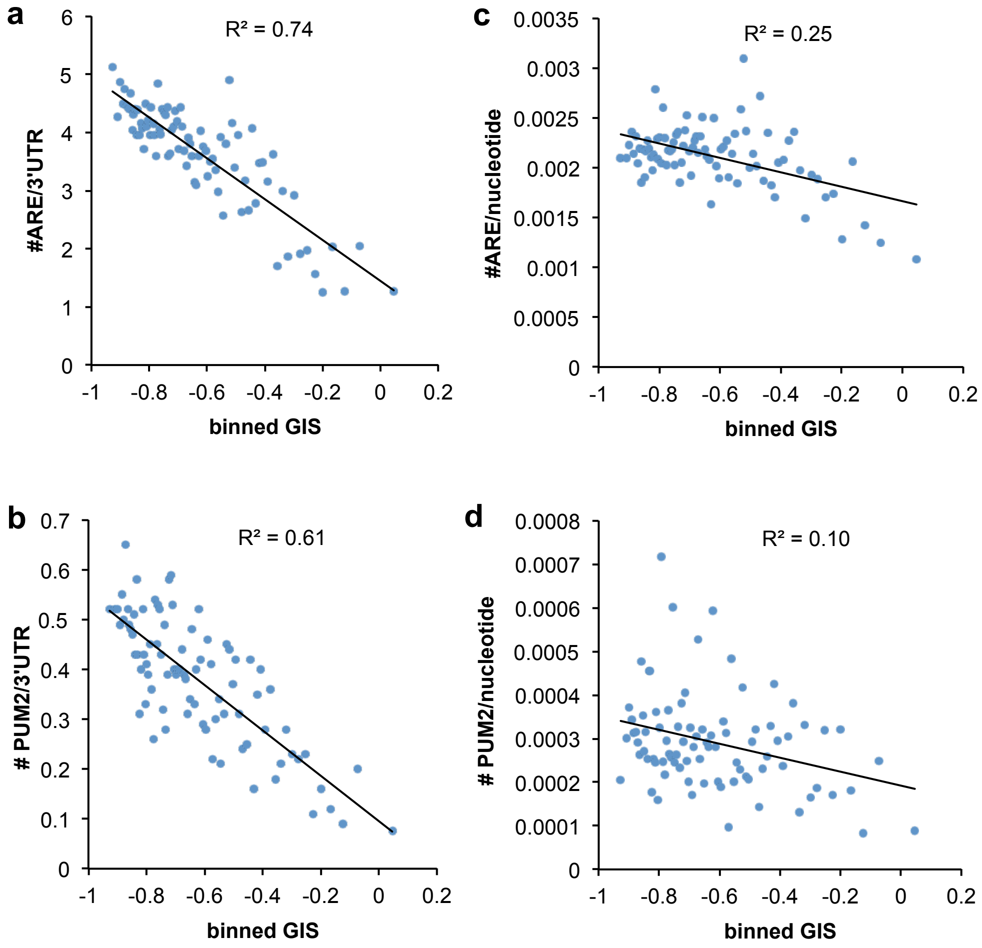

Supplementary Figure 8: Correlation of GIS with sequence motifs.
a,b, The average occurrences of ARE (**a**) and PUM2 motif sites (**b**) in bins of 100 genes are plotted against the average GIS in each bin. **c,d**, Similar to (**a** and **b**), but the density of motif sites is shown, since unstable transcripts are in general associated with longer 3´ UTRs.
